# Uptake of L-proline by Cultured Primary Rat Astrocytes

**DOI:** 10.1007/s11064-026-04886-5

**Published:** 2026-09-30

**Authors:** Paul Spellerberg, Ralf Dringen

**Affiliations:** 1 Faculty 2 (Biology/Chemistry), University of Bremen, Bremen, Germany; 2 Centre for Biomolecular Interactions Bremen, University of Bremen, P.O. Box 330440, 28334 Bremen, Germany

**Keywords:** Active transport, ASCT, Astrocytes, Proline, Uptake

## Abstract

The proteinogenic amino acid L-proline can be efficiently metabolized by cultured astrocytes as exogenous substrate to fuel mitochondrial ATP regeneration. To investigate the properties of the transport processes involved in astrocytic L-proline uptake, we used primary rat astrocyte cultures as model system. After application of L-proline, cultured astrocytes efficiently accumulated the amino acid in a time- and concentration-dependent saturable manner. Omission of sodium ions drastically lowered cellular L-proline accumulation to low values that increased proportional to the concentration of L-proline applied. The saturable sodium-dependent L-proline accumulation followed apparent Michaelis-Menten kinetics with a calculated K_M_ value of around 1.0 mM and a V_max_ value of around 20 nmol/(5 min x mg). In contrast to L-proline, D-proline was hardly taken up by the cells. L-Proline uptake was strongly affected by lowering the incubation temperature, while alterations of the extracellular pH value did not affect the accumulation of L-proline. An excess of the amino acids L-alanine, L-asparagine, L-methionine, L-serine, L-threonine or L-valine severely lowered the uptake of L-proline. Inhibition of uptake was also found in the presence of L-4-hydroxyproline and L-homocysteine as well as in the presence of the ASCT1 inhibitor L-4-chlorophenylgycine, but not after exposure to inhibitors of other potential astrocytic L-proline transporters. These data suggest that L-proline uptake by cultured rat astrocytes is mainly mediated by the sodium-dependent neutral amino acid transporter ASCT1.

**Supplementary Information:**

The online version contains supplementary material available at https://doi.org/10.1007/s11064-026-04886-5.

## Introduction

L-Proline is the only proteinogenic amino acid that has as special structural feature a secondary amino group [1]. L-Proline is important for cellular protein synthesis, but is also involved in cell signaling and in the modulation of neurotransmission [1–6]. For the brain, elevated L-proline concentrations have been reported to induce excitotoxicity [6] and oxidative stress [7]. Accordingly, disturbances of L-proline metabolism in the brain have been connected with neurological and psychiatric disorders [6, 8, 9].

Astrocytes are important glial cells in the brain that have essential functions in the ion and neurotransmitter homeostasis and play a major role in the energy, defense and amino acid metabolism of the brain [10–19]. L-proline has been shown to be metabolized by cultured astrocytes to glutamate that is used for GSH synthesis [20] and to glutamine that can be exported from the cells [21]. Extracellular L-proline has recently also been shown to be an excellent energy substrate for starved astrocytes [22, 23]. As shown for other cell types [4, 24], the mitochondrial flavoenzyme proline dehydrogenase (proline oxidase) catalyzes the first step of astrocytic L-proline catabolism [22, 23].

A prerequisite for the consumption of L-proline by astrocytes is the cellular uptake of the amino acid. Of the many different types of amino acid transporters [25, 26], several have been reported to accept L-proline as a substrate [25, 27–35]. Of these L-proline transporters, 5 have been reported to be expressed in astrocytes (Table 1). Other transporters that have been connected with L-proline transport in brain cells are the proton-dependent PAT1/2/4 (SLC38A1/2/4) [27] and the sodium-dependent high affinity proline transporter PROT (SLC6A7) [36, 37], but these transporters are mainly expressed in neurons and not in astrocytes [37–41].

**Table 1:** Properties of amino acid transporters that are expressed in astrocytes and have been reported to accept L-proline as substrate

Transporter	ASCT1	B^0^AT2 (SBAT1)	Imino (SIT1)	ATA2 (SNAT2)	SNAT5
Gene	SLC1A4	SLC6A15	SLC6A20	SLC38A2	SLC38A5
Expression in astrocytes	Yes [30, 38, 59, 63, 67–71]	Yes [72]	Yes [31]	Yes [35, 38, 70, 73, 74] No [33]	Yes [75]
Substrates					
L-Proline	Yes [25, 28, 76–78]	Yes [25, 27, 35, 79]	Yes [25, 27, 29]	Yes [27, 80, 81]	Yes [82] No [81, 83]
Proteinogenic amino acids	A, C, N, P, S, T, V [25, 27, 28, 76–78]	A, C, F, I, L, M, N, P, Q, S, T, V [25, 27, 35, 79]	G, P [27, 29, 31]	A, C, G, H, M, N, P, Q, S [27, 80, 81]	A, C, F, G, H, I, M, N, P, Q, S, T, V, Y [82]
Other amino acids	HP [28, 68] D-Ser [59, 68, 84] HC [64, 65]	HP [79]	HP [29, 85] Sarcosine [29]	?	?
Sodium dependency	Yes [27, 30, 76, 78, 86–88]	Yes [35, 79]	Yes [29]	Yes [80]	Yes [81]
Proton dependency	No [89]	No [79]	No [29]	No [33]	Yes [81]
K_M_ values for L-proline (µM)	704 ± 86 [28]	195 ± 16 [35] 380 ± 20 [79]	170 ± 50 [29] 130 ± 30 [85]	Around 800 [25, 33]	?
Stereospecificity for L-proline	?	Yes [79]	Yes [29]	Yes [90]	?
Frequently used inhibitor	CPG [66]	Loratadine [91]	MeAIB [29, 85]	MeAIB [80]	L-glutamic acid γ-monohydroxamate [92]

Proteinogenic amino acid substrates are given in the one-letter code

*CPG* L-4-chlorophenylglycine, *HC *L-homocysteine, *HP* 4-hydroxy-L-proline, *MeAIB *N-methylaminoisobutyric acid; ? = no information found

Although astrocytes are well known to metabolize L-proline, the transporter(s) that may contribute to the L-proline uptake in astrocytes have to our knowledge not been identified so far. In order to elucidate the transport mechanisms that contribute to the astrocytic uptake of L-proline, we investigated the cellular accumulation of L-proline by cultured primary astrocytes after inhibition of cellular L-proline catabolism. Our results demonstrate that astrocytes accumulate L-proline in a time- and concentration-dependent manner in a process that strongly depends on the presence of sodium ions and on the temperature. L-Proline uptake was found to be stereospecific for the L-isomer of proline and not affected by changes in the extracellular pH. An excess of the amino acids L-alanine, L-asparagine, L-methionine, L-serine, L-threonine or L-valine severely lowered the uptake of L-proline as did the presence of L-4-hydroxyproline or L-homocysteine as well as the presence of the ASCT1 inhibitor L-4-chlorophenylgycine. These data suggest that L-proline uptake into astrocytes is mainly mediated by ASCT1.

## Materials and Methods

### Materials and Chemicals

Sterile cell culture materials and microtiter plates were obtained from Sarstedt (Nümbrecht, Germany). Fetal calf serum, penicillin G/streptomycin sulfate solution and Dulbecco’s modified Eagle´s medium (DMEM) were purchased from Thermo Fisher Scientific (Schwerte, Germany). L-Proline, L-asparagine, L-aspartate, L-trans-4-hydroxyproline and L-phenylalanine were from Fluka (Buchs, Switzerland). L-Alanine, L-glycine, L-glutamate, L-glutamine, L-isoleucine, L-lysine, L-serine, L-threonine, L-tryptophane, L-homocysteine, D-proline, D-glucose, α-methylaminoisobutyric acid (MeAIB), sarcosine, L-4-chlorophenylglycine (CPG), KCl, NaCl, glacial acetic acid and ninhydrin were from Sigma Aldrich (Steinheim, Germany). 4-(2-Hydroxyethyl) piperazine-1-ethanesulfonic acid (HEPES) and K_2_HPO_4_ were from Carl Roth (Karlsruhe, Germany). CaCl_2_, KOH, Na_2_HPO_4_ and pyruvate were obtained from Merck (Darmstadt, Germany) and NaOH was from VWR (Darmstadt, Germany). L-Arginine, L-histidine, L-leucine, L-methionine, L-valine, L-tyrosine, MgCl_2_, choline chloride, NADH, dimethyl sulfoxide (DMSO) and bovine serum albumin (BSA) were obtained from AppliChem (Darmstadt, Germany). (S)-(-)-Tetrahydro-2-furoric acid (THFA) was purchased from Santa Cruz Biotechnology (Dallas, TX, USA), (S)-(+)−5-oxotetrahydrofuran-2-carboxylic acid (5-Oxo) was from Tokyo Chemical Industry (Tokyo, Japan) and N-propargylglycine (N-PPG) and loratadine were from MedChem Express (Monmouth Junction, NJ, USA).

### Primary Astrocyte Cultures

Primary cultures of astrocytes were prepared as previously described [42] from the whole brains of neonatal Wistar rats. Rats for mating had been purchased from Charles River Laboratories (Sulzfeld, Germany) and were treated according to the State of Bremen, German and European animal welfare acts. The harvested cells were suspended in culture medium (90% DMEM (25 mM glucose, 44.6 mM sodium bicarbonate, 1 mM pyruvate, 20 U/mL penicillin G, 20 µg/mL streptomycin sulfate) and 10% fetal calf serum) and 1 mL of the suspension was seeded into wells of 24-well plates. The cell cultures were incubated in a humidified Sanyo cell incubator (Osaka, Japan) at 37 °C and with 10% CO_2_. The culture medium was renewed every seventh day and one day prior to experiments. Experiments were conducted on confluent astrocyte cultures of an age between 14 and 28 days. Primary rat astrocyte cultures are strongly enriched for astrocytes and contain only low numbers of other glia cell types [42–44].

### Experimental Incubations

For experimental incubations, the cells were washed twice with 1 mL of warm (37 °C) washing buffer (145 mM NaCl, 20 mM HEPES, 5.4 mM KCl, 1.8 mM CaCl_2_, 1 mM MgCl_2_ and 0.8 mM Na_2_HPO_4_; adjusted with NaOH to pH 7.4 at 37 °C) before they were pre-incubated in 250 µL pre-incubation buffer (PB; 3 mM glucose, 1 mM of the proline dehydrogenase inhibitor N-propargylglycine (N-PPG) [22, 45], 145 mM NaCl, 20 mM HEPES, 5.4 mM KCl, 1.8 mM CaCl_2_, 1 mM MgCl_2_ and 0.8 mM Na_2_HPO_4_; adjusted with NaOH to pH 7.4 at 37 °C) for 60 min at 37 °C in a cell incubator without CO_2_ supply. Subsequently, the PB was removed and the cells were washed twice with 1 mL of the glucose- and N-PPG-free versions of the incubation buffers (IBs) used for the following main incubation to study proline accumulation (IB; 3 mM glucose, 1 mM N-PPG, 145 mM NaCl, 20 mM HEPES, 5.4 mM KCl, 1.8 mM CaCl_2_, 1 mM MgCl_2_ and 0.8 mM Na_2_HPO_4_; pH adjusted to 7.4 (if not stated otherwise) with NaOH at the respective incubation temperature). For the preparation of Na^+^-free IB, the NaCl was substituted with choline chloride, the Na_2_HPO_4_ was substituted with K_2_HPO_4_ and the pH was adjusted with KOH. Cellular proline accumulation was investigated by incubation of the pre-incubated cells at 37 °C (if not stated otherwise) in 250 µL of the given glucose and N-PPG-containing IBs that contained proline and other substances in the concentrations indicated in the legends of the figures and tables. After the given incubation times, the media were collected and used to determine extracellular activities of lactate dehydrogenase (LDH). The cells were washed twice with 1 mL ice-cold phosphate-buffered saline (PBS; 10 mM potassium phosphate buffer, 150 mM NaCl; pH 7.4) and lysed for determination of the cellular proline content. The N-PPG stock solution was dissolved in DMSO, leading to a final DMSO concentration of 1% (v/v) in the PB and IBs.

### Determination of the Cellular Proline Content

The accumulation of L-proline by astrocytes was determined by a colorimetric method that uses the quantification of the proline-ninhydrin product formed under acidic conditions at high temperature [46] as previously described [23]. The washed cells were lysed in 120 µL glacial acetic acid and 100 µL of the lysate were mixed with 200 µL ninhydrin solution (1:1 mixture of glacial acetic acid and IB containing 1.25% (w/v) ninhydrin). After incubation at 100 °C for 30 min, the reaction tubes were rapidly cooled down on ice and 200 µL samples were transferred into wells of a microtiter plate. The absorbance spectrum of the reaction products was determined and the absorbance measured at 514 nm was used for proline quantification by comparison of the absorbance values to those of a linear calibration curve (Fig. S1) obtained from the absorbances determined for L-proline calibration standards (0 to 50 µM L-proline in IB). For determination of the cellular D-proline accumulation, calibration standards were prepared using D-proline, which gave almost identical absorbances as L-proline (Fig. S1). For the calculation of the cellular L-proline accumulation, the apparent cellular L-proline content determined after the 1 h pre-incubation period was subtracted from the values obtained after the respective main incubations.

### Determination of Cell Viability and Initial Protein Content

Cell viability was assessed by determining the extracellular LDH activity as an indicator for impaired membrane integrity. LDH activity was measured as previously described [42], utilizing the LDH-mediated oxidation of NADH to NAD^+^ in the presence of pyruvate that was monitored at 340 nm. The extracellular LDH activity is given in percent of the initial cellular LDH activity. Initial protein contents were determined by the Lowry method [47], using bovine serum albumin as standard.

### Data Presentation and Statistical Analysis

If not stated otherwise, the data shown represent means ± standard deviation (SD) of values obtained in three independent experiments that had been performed in duplicate (triplicate for initial values) wells of independently prepared cultures. For statistical analysis, the normal distribution of residuals was assessed via quantile-quantile plots with a 95% confidence interval [48]. Statistical significance of differences between two data sets was analyzed by a paired, two-tailed t-test. Analysis of statistical significance of differences between three or more data sets was performed by Analysis of Variance (ANOVA) followed by the Bonferroni post-hoc test. The levels of significance of differences are indicated by ^#/^*^/+^*p* < 0.05; ^##/^**^/++^*p* < 0.01 and ^###/^***^/+++^*p* < 0.001. *p* > 0.05 was considered as not significant (ns).

## Results

### Concentration- and Time-Dependent Uptake of L-Proline by Astrocytes

Primary astrocytes have recently been reported to consume L-proline as energy substrate [22, 23] which requires efficient uptake of this amino acid into the cells. To investigate the mechanisms involved in the uptake of L-proline, astrocyte cultures were incubated with L-proline after a preincubation with the proline dehydrogenase inhibitor N-PPG [22, 45] to prevent cellular metabolism of the internalized L-proline. For the given incubations, the cellular L-proline content was quantified by a colorimetric method making use of the proline-ninhydrin complex formed under acidic conditions at high temperature [22, 23, 46]. For untreated astrocytes, the ninhydrin complexes formed by cellular components resulted in a basal absorbance signal that accounted for an apparent initial cellular proline content of 5.3 ± 0.9 nmol/mg (data from 32 independent experiments performed on 20 astrocytes cultures). However, these complexes revealed spectral characteristics that are different from those of the proline-ninhydrin complex and, accordingly, also the typical ratio of the absorbances (1.4) measured for the two peaks in the absorption spectrum (480 nm and 514 nm) of the ninhydrin-L-proline complex (Fig. S1) was not found for untreated cells (data not shown), demonstrating that a substantial part of the ninhydrin complex signals obtained for untreated cells did not represent cellular proline. Most amino acids do not react with ninhydrin under the acidic and high temperature assay conditions used to complexes that absorb at 514 nm (Fig. S1). However, cysteine and ornithine have been reported to react under the assay conditions used with ninhydrin [49] and may contribute together with some initial cellular proline to the low basal absorbance that was found for cultured astrocytes before application of L-proline.

**Fig. 1:**
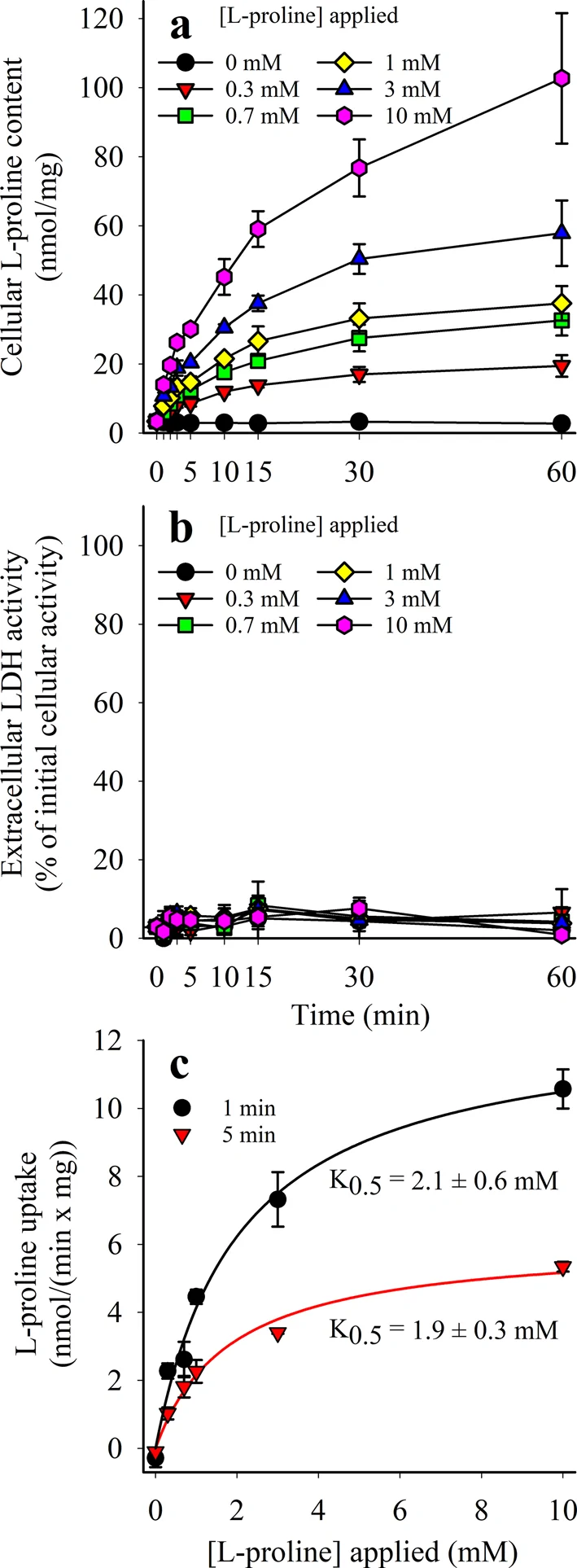
Time-dependent uptake of L-proline by cultured astrocytes. The cells were pre-incubated with N-PPG for 1 h to inhibit L-proline metabolism and then incubated with the given L-proline concentrations for the indicated time periods. For the given incubations the cellular L-proline contents (**a**) and the extracellular LDH activities (**b**) were measured. The data obtained for incubations of 1 min or 5 min were used to calculate the specific uptake velocities for the L-proline accumulation by astrocytes (**c**). The calculated applied L-proline concentrations at which half-maximal uptake velocities were reached (K_0.5_) are given (**c**). The apparent initial cellular L-proline content before the preincubation was 4.6 ± 0.3 nmol/mg, the initial cellular LDH activity was 105 ± 43 nmol/(min x well) and the initial protein content was 121 ± 21 µg/well. The data represent means ± SD of values obtained in three independent experiments that had been performed on independently prepared cultures

During the 60 min pre-incubation with N-PPG and during a 60 min main incubation without L-proline the signals for the apparent cellular proline content declined yielding apparent specific proline contents of 3.7 ± 0.7 nmol/mg (Table 2) and 2.6 ± 0.4 nmol/mg (Fig. 1a), respectively. For all experiments performed to investigate the cellular accumulation of L-proline by astrocytes, the apparent cellular proline content found after the pre-incubation was subtracted from the values obtained after an exposure to L-proline under the given incubation conditions to calculate the real cellular L-proline accumulation velocity.

**Table 2:** L-proline content of astrocytes that had been treated with 0.2 mM or 1 mM L-proline

[L-proline] (mM)	Incubation time (min)	Specific cellular L-proline content (nmol/mg)	Uptake velocity (nmol/(5 min x mg))	LDH release (% of initial cellular LDH activity)	Number of experiments (number of independent cultures)
0	0	3.7 ± 0.7	0	4 ± 4	32 (20)
0.2	5	7.5 ± 1.0	3.9 ± 1.2	3 ± 3	33 (20)
1	5	14.3 ± 2.4	10.8 ± 2.4	4 ± 3	18 (9)

Primary astrocytes were pre-incubated with N-PPG for 1 h, followed by a 5 min main incubation with either 0.2 mM or 1 mM L-proline. After the pre-incubation (0 min) and after the 5 min main incubation, the cellular L-proline contents were measured and used to calculate the uptake velocities. Additionally, the extracellular LDH activities were measured. The data shown represent the means ± SD of values determined in n independent experiments performed on the given number (in brackets) of independently prepared cultures

To study basal parameters of L-proline accumulation, cultured astrocytes were incubated in the absence or the presence of L-proline in concentrations of up to 10 mM for up to 60 min before the cellular content of L-proline was quantified. Already the application of 0.3 mM L-proline led to a strong increase in the cellular L-proline content and almost constant levels of L-proline were observed after incubations for 30–60 min (Fig. 1a). Increasing the extracellular L-proline concentration accelerated the uptake and the cellular accumulation of L-proline. After 60 min of incubation with L-proline in initial concentrations of 0.3 mM and 10 mM, the cellular L-proline content accounted for of 17.7 ± 4.6 nmol/mg and 92.5 ± 21.3 nmol/mg, respectively. Calculation of the accumulated cytosolic L-proline concentrations for 60 min incubations by making use of the known cytosolic volume of cultured astrocytes of 4.1 µL/mg [50] revealed that the accumulated cytosolic L-proline concentrations accounted for 4.3 ± 1.1 mM (0.3 mM applied), 7.2 ± 1.8 mM (0.7 mM applied), 8.3 ± 1.8 mM (1 mM applied), 12.7 ± 2.3 mM (3 mM applied) and 22.6 ± 5.2 mM (10 mM applied). None of the experimental conditions applied caused any significant increase in extracellular LDH activity during the 60 min incubations (Fig. 1b), demonstrating that the conditions used did not compromise cell viability.

For all concentrations of L-proline applied, the very rapid initial cellular accumulation of L-proline slowed down over time towards reaching a plateau of cellular L-proline content (Fig. 1a). As a consequence, the analysis of the maximal uptake velocity for L-proline uptake was affected by the incubation time that was chosen to obtain the data for the analysis. For incubation periods of both 1 min or 5 min, the L-proline uptake into primary astrocytes was concentration-dependent and followed apparent Michaelis-Menten type kinetics (Fig. 1c). The calculated V_max_ values were 12.8 ± 1.1 nmol/(min x mg) for 1 min incubations and 6.1 ± 0.4 nmol/(min x mg) for 5 min incubations. However, calculation of the L-proline concentrations that enabled the cells to accumulate L-proline with half-maximal uptake velocity (K_0.5_) were almost identical for both incubation periods analyzed, with 2.1 ± 0.6 mM (calculated for 1 min incubations) and 1.9 ± 0.3 mM (calculated for 5 min incubations).

For most of the further experiments to characterize the mechanisms involved in the analysis of L-proline uptake, primary astrocytes were incubated for 5 min with either 0.2 mM or 1 mM L-proline to enable the cells to accumulate L-proline to values that were sufficiently high for a reliable analysis of the uptake process after subtraction of the apparent basal L-proline content of pre-incubated astrocytes. Compiled data from a large number of independent experiments revealed that the cellular L-proline contents increased within 5 min from an average initial apparent content after the pre-incubation (3.7 ± 0.7 nmol/mg), for incubations with 0.2 mM L-proline to 7.5 ± 1 nmol/mg and for incubations with 1 mM L-proline to 14.3 ± 2.4 nmol/mg, corresponding to average uptake velocities of 3.87 ± 1.15 nmol/(5 min x mg) for 0.2 mM L-proline and 10.8 ± 2.4 nmol/(5 min x mg) for 1 mM L-proline (Table 2).

### Tests for the Na^+^-, pH- and Temperature-Dependency of the L-Proline Uptake by Astrocytes

**Fig. 2:**
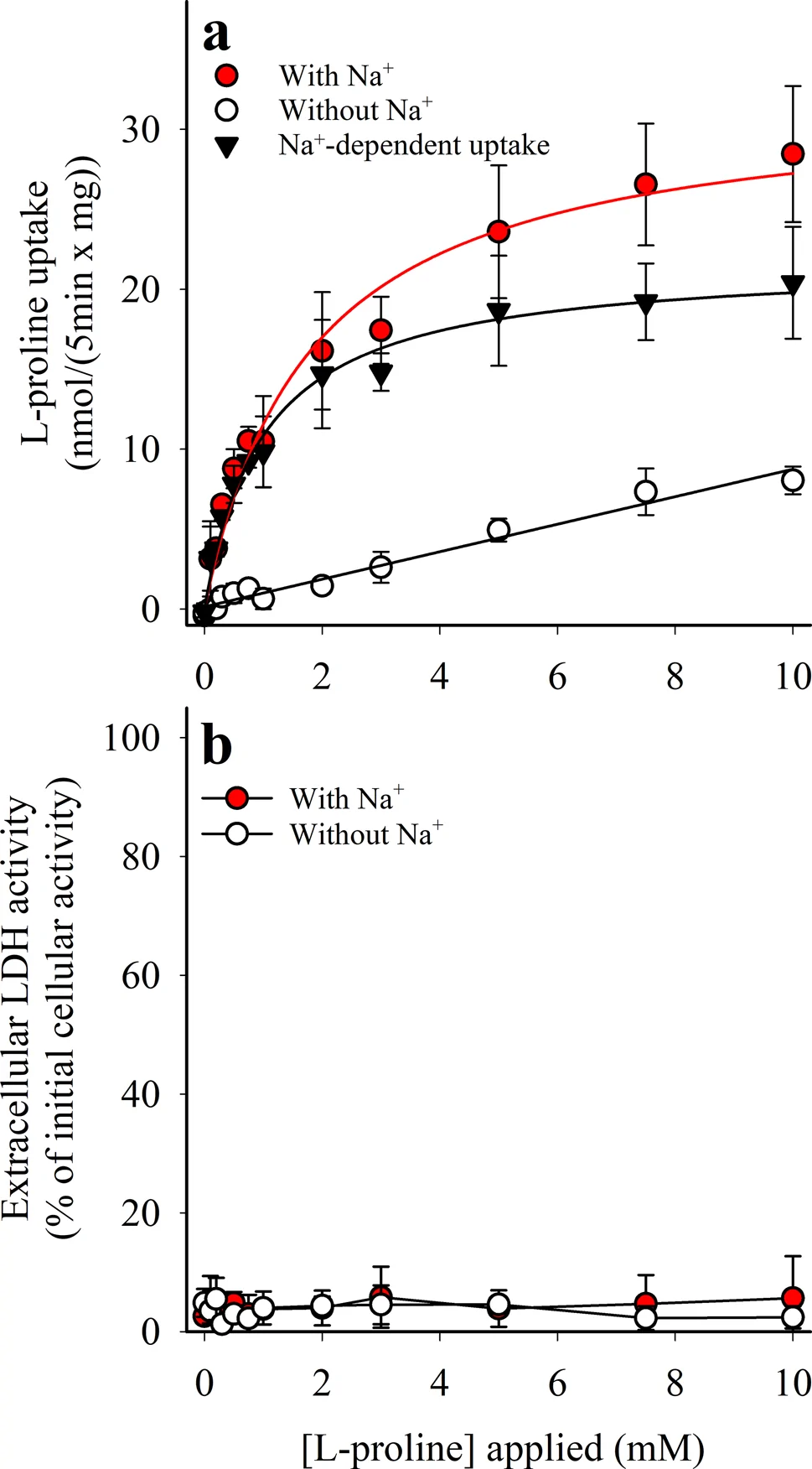
Na^+^-dependent uptake of L-proline by cultured astrocytes. Primary astrocytes were pre-incubated in Na^+^-containing PB with N-PPG for 1 h, followed by a 5 min main incubation in Na^+^-containing or Na^+^-free IBs that contained the indicated L-proline concentrations. After the incubation, the cellular L-proline contents were determined and used to calculate specific L-proline uptake velocities (**a**). In addition, the extracellular LDH activities were measured (**b**). For each L-proline concentration applied, the values for the uptake determined after incubation in the presence of Na^+^ were corrected for the values determined for the uptake in the absence of Na^+^ to obtain data for the Na^+^-dependent L-proline uptake (**a**). The initial cellular LDH activity was 129 ± 46 nmol/(min x well) and the initial protein content was 139 ± 22 µg/well. The data represent means ± SD of values obtained in three independent experiments that had been performed on independently prepared cultures

Many of the known L-proline transporters are Na^+^-dependent (Table 1). In order to investigate whether the L-proline accumulation by astrocytes depends on the presence of Na^+^, cultured astrocytes were incubated for 5 min in the absence or the presence of L-proline in concentrations of up to 10 mM in either Na^+^-containing or Na^+^-free IB. The concentration-dependency of L-proline accumulation in the presence of Na^+^ followed a hyperbolic function, but a clear maximal value was not reached (Fig. 2a). In contrast, L-proline accumulation in the absence of Na^+^ was much lower than the accumulation found in the presence of Na^+^ and increased for higher L-proline concentrations proportional to the concentration of L-proline applied with a slope of 0.86 nmol/(5 min x mg x mM) (Fig. 2a). Calculation of the Na^+^-dependent L-proline accumulation by subtracting the values determined after incubation with and without Na^+^ revealed a hyperbolic Michaelis-Menten type concentration-dependency (Fig. 2a) with a maximal uptake rate of 21.8 ± 3.8 nmol/(5 min x mg) and a half-maximal uptake velocity found for an initial L-proline concentration of 1.0 ± 0.19 mM. None of the conditions applied caused any significant increase in extracellular LDH activity (Fig. 2b).

**Fig. 3:**
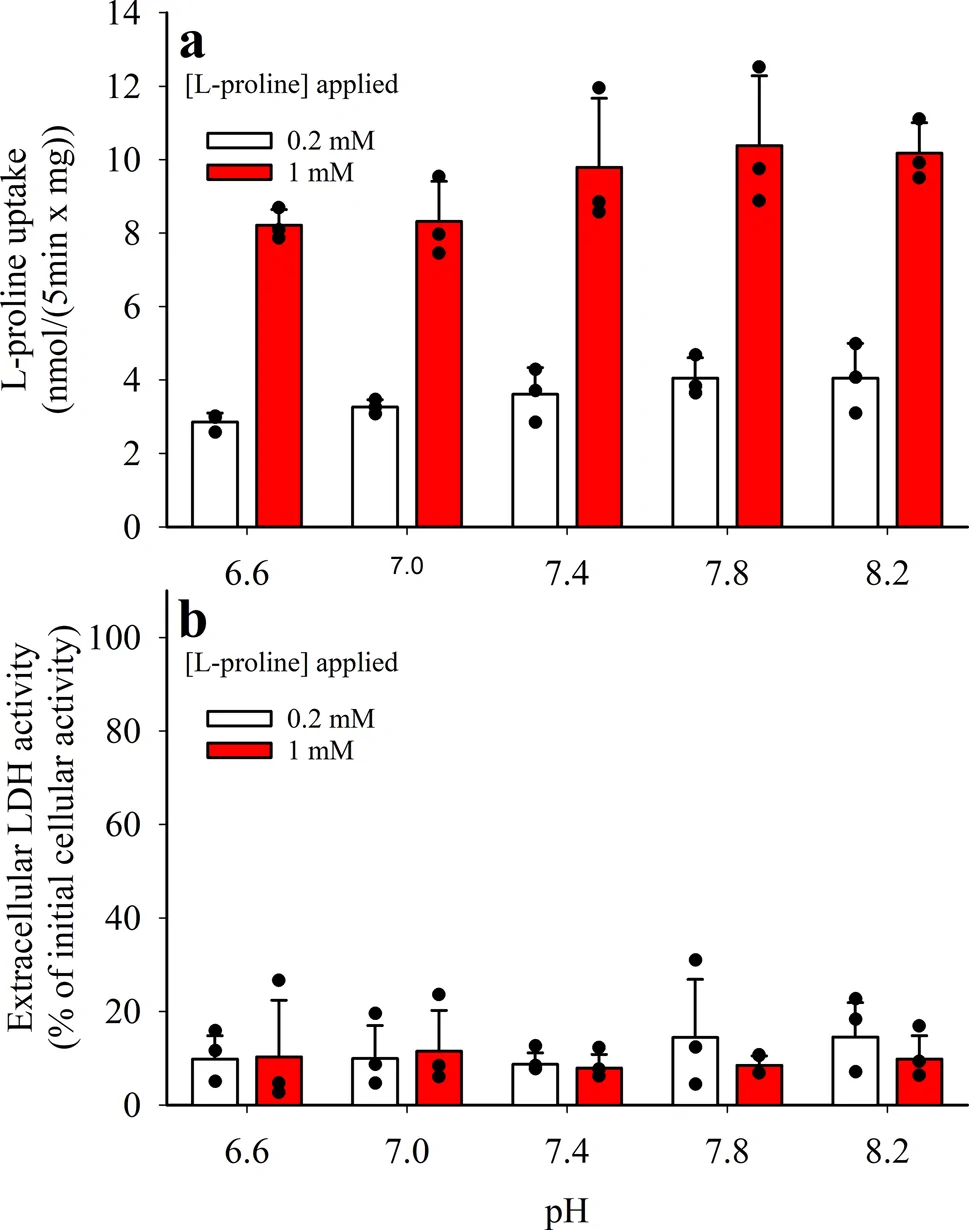
Test for potential effects of pH on the uptake of L-proline by cultured astrocytes. The cells were pre-incubated in PB (pH 7.4) with N-PPG for 1 h, followed by a 5 min main incubation in IBs (adjusted to the given pH values) with either 0.2 mM or 1 mM L-proline. After the incubation, cellular L-proline levels were determined and used to calculate specific L-proline uptake velocities (**a**). Additionally, the extracellular LDH activities were determined (**b**). The initial cellular LDH activity was 113 ± 25 nmol/(min x well) and the initial protein content was 127 ± 18 µg/well. The data represent means ± SD of values obtained in three independent experiments that had been performed on independently prepared cultures. Statistical analysis (ANOVA) revealed no significant impact of the pH on the uptake of L-proline compared to the incubation at pH 7.4

To test whether the extracellular proton concentration may affect the accumulation of L-proline by astrocytes, the cells were incubated with 0.2 mM or 1 mM L-proline in incubation buffers that maintained extracellular pH values ranging from pH 6.6 to pH 8.2. Variation of the extracellular pH did not significantly affect the astrocytic L-proline accumulation (Fig. 3a) compared to the respective control incubation (pH 7.4), nor was the cell viability affected by these treatments (Fig. 3b).

**Fig. 4:**
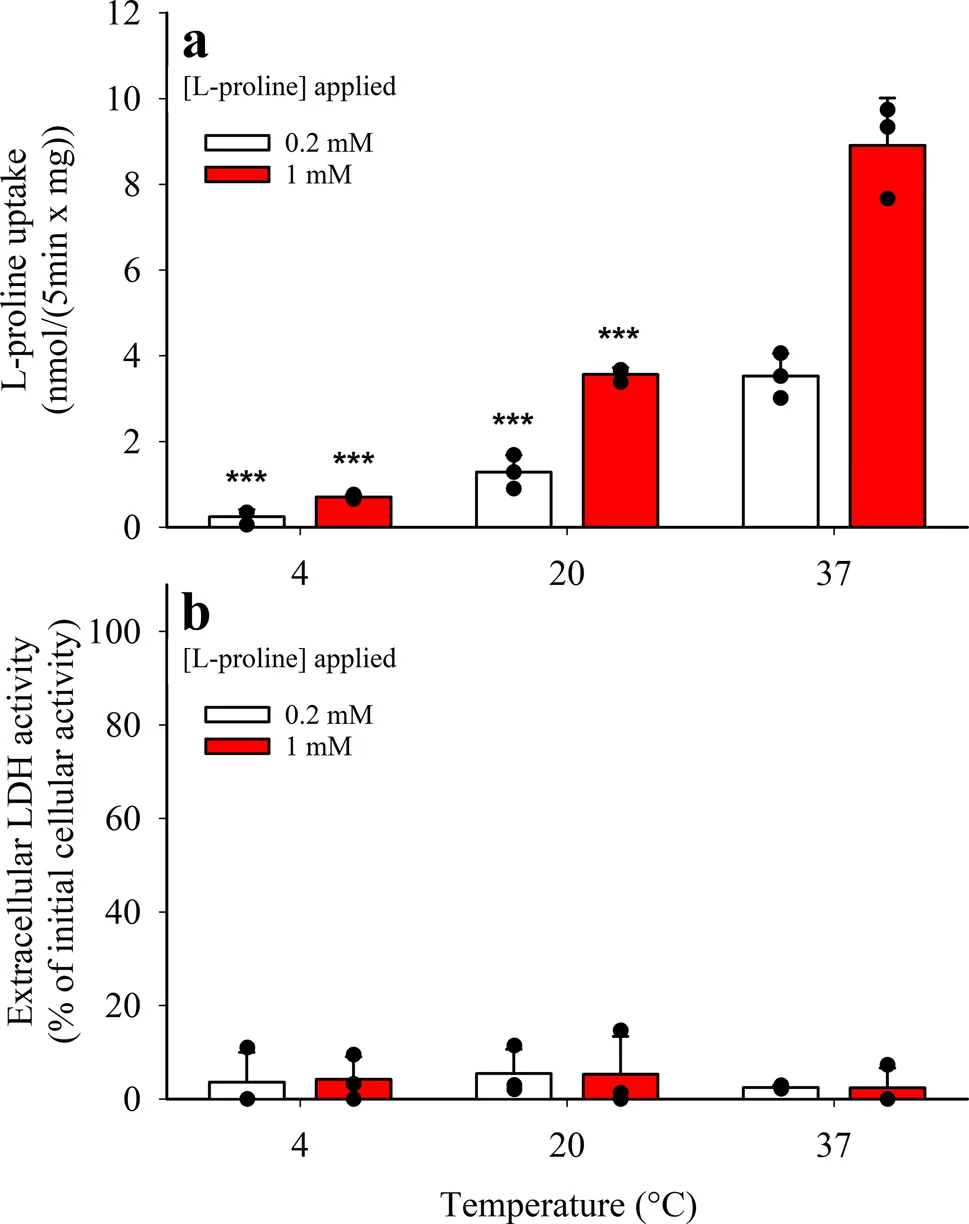
Test for the effect of temperature on the uptake of L-proline by cultured astrocytes. The cells were pre-incubated in PB with N-PPG for 1 h, followed by a 5 min main incubation with either 0.2 mM or 1 mM L-proline at the given temperatures. After the incubation, cellular proline levels were determined and used to calculate specific proline uptake velocities (**a**). Additionally, the extracellular LDH activities were determined (**b**). The initial cellular LDH activity was 80 ± 4 nmol/(min x well) and the initial protein content was 109 ± 1 µg/well. The data represent means ± SD of values obtained in three independent experiments that had been performed on independently prepared cultures. The significance of differences of data (ANOVA) compared to the respective data obtained for incubations at 37 °C is indicated as ***p < 0.001

In order to assess the effect of the incubation temperature on the L-proline uptake, primary astrocytes were incubated with 0.2 mM or 1 mM L-proline in incubation buffers that had been adjusted to pH 7.4 at temperatures of 4 °C, 20 °C or 37 °C. Compared to an incubation at 37 °C, the uptake of L-proline into astrocytes was strongly and significantly lowered by incubations at 20 °C and 4 °C by around 40% and 90%, respectively, for both L-proline concentrations applied (Fig. 4a). The cell viability was not affected by the incubations at the given temperatures (Fig. 4b).

### Test for the Stereospecificity of Proline Uptake into Primary Astrocytes

**Fig. 5:**
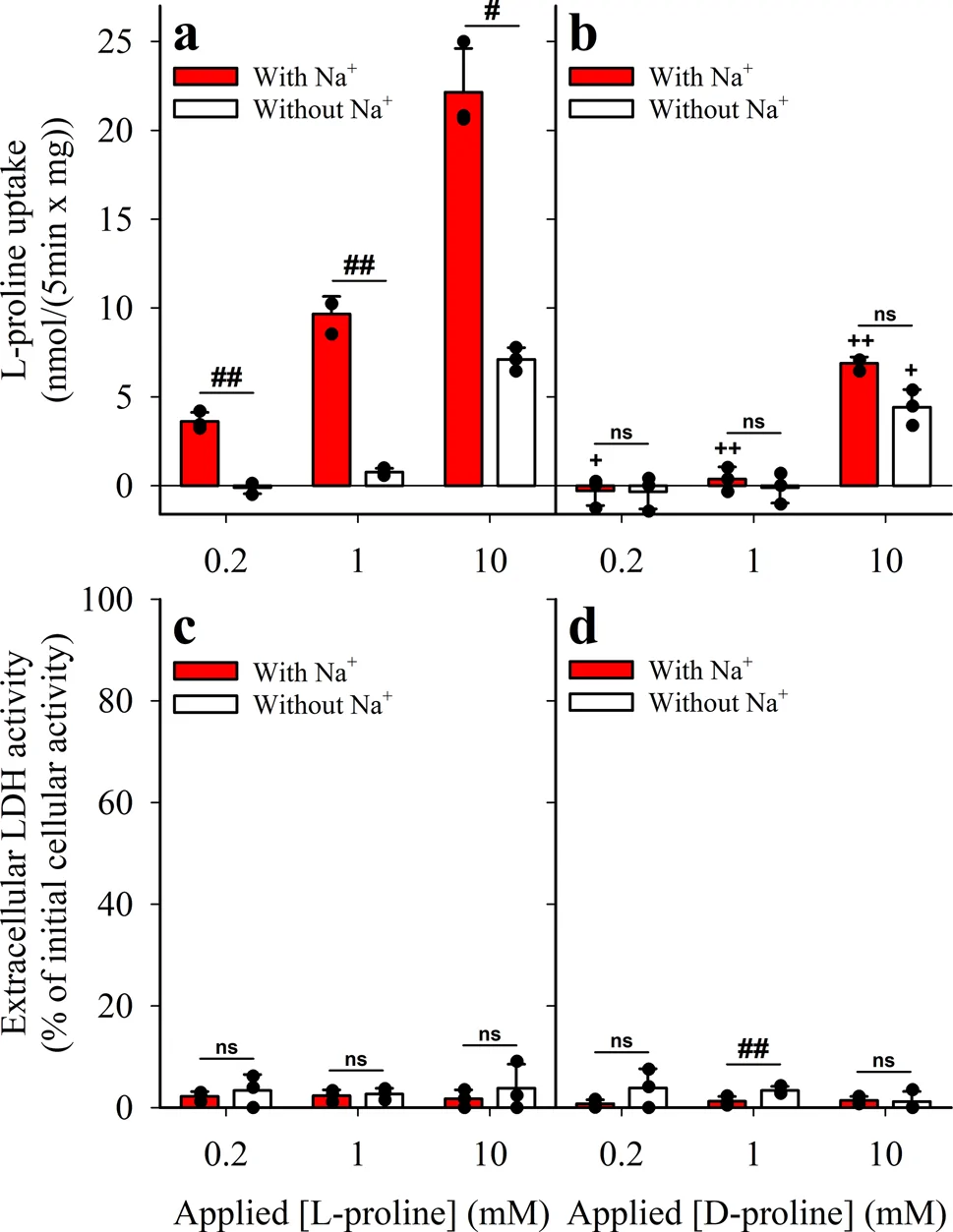
Test for the stereospecificity of the uptake of proline by cultured astrocytes. The cells were pre-incubated in Na^+^-containing PB with N-PPG for 1 h, followed by a 5 min main incubation in Na^+^-containing or Na^+^-free IBs with the indicated concentrations of either L-proline or D-proline. After the incubation, the cellular proline levels were determined and used to calculate specific proline uptake velocities of either L-proline (**a**) or D-proline (**b**). In addition, the extracellular LDH activities were determined (c, d). The initial cellular LDH activity was 117 ± 11 nmol/(min x well) and the initial protein content was 134 ± 5 µg/well. The data represent means ± SD of values obtained in three independent experiments that had been performed on independently prepared cultures. Significance of differences (paired t-test) is indicated for the comparison of data obtained from incubations of one stereoisomer in the absence or the presence of Na^+^ (^#^*p* < 0.05; ^##^*p* < 0.01) and for the comparison of data from the respective incubations with L- and D-proline (^+^*p* < 0.05, ^++^ *p* < 0.01) (ns = not significant)

Primary astrocytes efficiently consume and utilize exogenous L-proline for ATP regeneration, but not its stereoisomer D-proline [22]. To test for the stereospecificity of proline uptake, primary astrocytes were incubated with 0.2 mM, 1 mM or 10 mM of either L- or D-proline in the absence or the presence of Na^+^. In the presence of Na^+^, L-proline was taken up into the cells in a concentration dependent-manner (Fig. 5a). In the absence of Na^+^, this accumulation was almost completely prevented for incubations with 0.2 mM or 1 mM L-proline and lowered by around 70% for an incubation with 10 mM L-proline (Fig. 5a). In contrast, after exposure to 0.2 mM or 1 mM D-proline, hardly any cellular D-proline was determined for incubations in the presence or the absence of Na^+^ (Fig. 5b), while for incubations with 10 mM D-proline around 5 nmol/(5 min x mg) were found accumulated for both the treatments in the absence and the presence of Na^+^ (Fig. 5b). The low extracellular LDH activity found for the incubation conditions applied (Fig. 5c, d) revealed that the cell viability was not impaired by the incubations with L- or D-proline.

### Effect of L-Amino Acids on the Astrocytic Accumulation of L-Proline

**Fig. 6:**
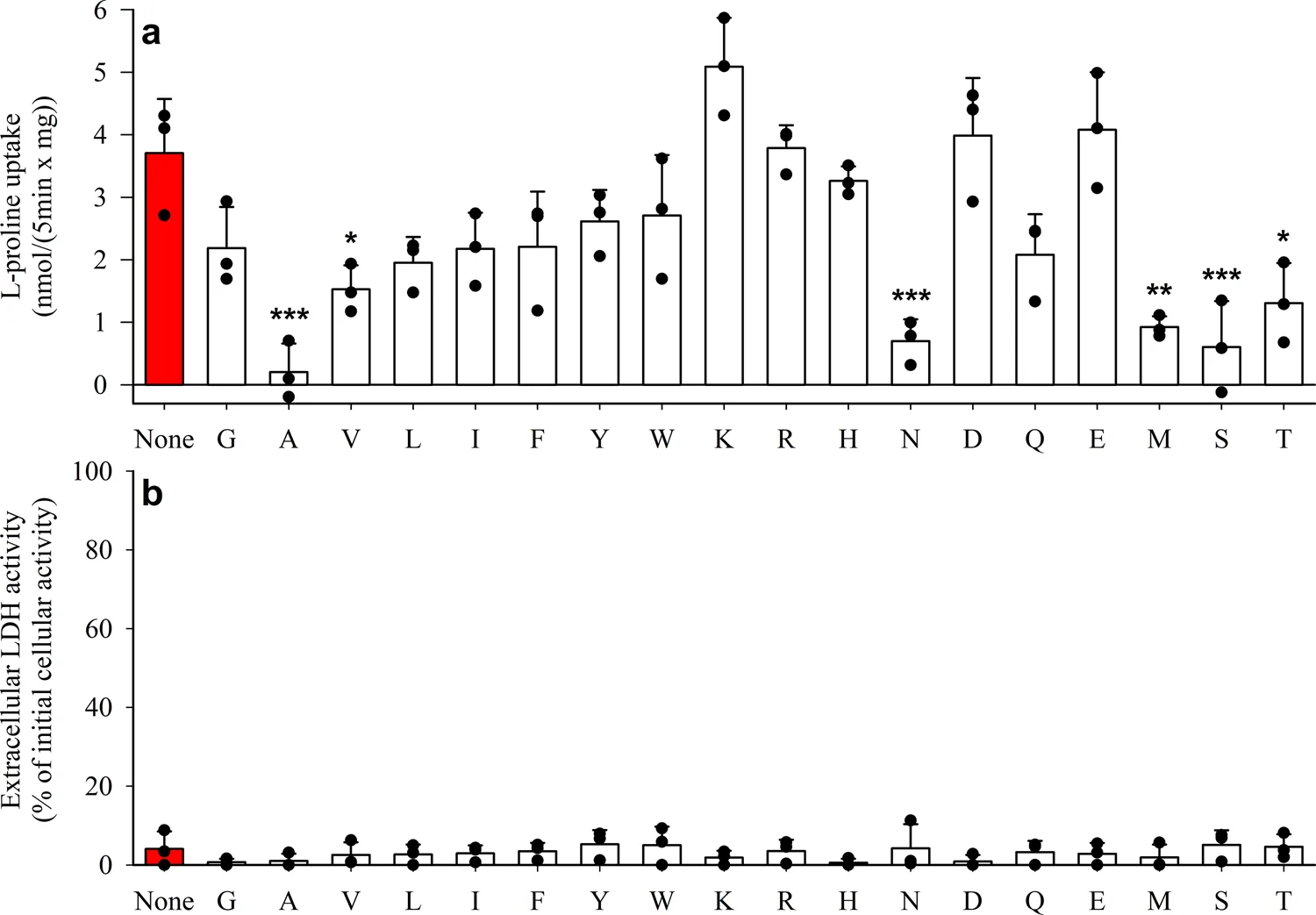
Test for the potential of proteinogenic amino acids to affect the astrocytic uptake of L-proline. Primary astrocytes were pre-incubated in PB with N-PPG for 1 h, followed by a 5 min main incubation with 0.2 mM L-proline in the absence (none) or the presence of 2 mM of the L-stereoisomers of the indicated amino acids. After the incubation, the cellular proline levels were determined and used to calculate the specific proline uptake velocities (**a**). Additionally, the extracellular LDH activities were measured (**b**). The initial cellular LDH activity was 132 ± 35 nmol/(min x well) and the initial protein content was 136 ± 29 µg/well. The data represent means ± SD of values obtained in three independent experiments that had been performed on independently prepared cultures. Significance of differences (ANOVA) is indicated for comparison to data obtained for incubations in the absence of an additional amino acid (None) as *p < 0.05, **p < 0.01 and ***p < 0.001

Most amino acid transporters are not specific for a single amino acid as substrate but rather transport a variety of amino acids although with differing efficiency [25–27]. To test for the potential of proteinogenic amino acids to lower cellular L-proline accumulation by competing for L-proline uptake, primary astrocytes were incubated for 5 min with 0.2 mM L-proline in the presence of a 10-fold excess of individual L-amino acids. Of the proteinogenic amino acids only L-cysteine was not tested as this amino acid in a concentration of 2 mM interfered strongly with the proline assay and generated high absorbances even in the absence of L-proline (data not shown). Of the 18 amino acids tested for their competitive potential regarding L-proline uptake, 6 amino acids were found that in a 10-fold excess significantly lowered the uptake of L-proline into primary astrocytes, namely L-alanine (by 96 ± 12%), L-asparagine (by 82 ± 6%), L-serine (by 86 ± 19%), L-methionine (by 73 ± 12%), L-threonine (by 66 ± 10%) and L-valine (by 57 ± 14%) (Fig. 6a). All other amino acids had no significant impact on the astrocytic L-proline accumulation (Fig. 6a). None of these incubations caused any significant increase in extracellular LDH activity compared to the control condition (none) (Fig. 6b; Table 3).

**Table 3:** Concentration-dependent inhibition of astrocytic L-proline uptake by amino acids and transport inhibitors

Inhibitor	EC_50_ (mM) as calculated from the curve fittings	Maximal inhibition of L-proline uptake (%) as calculated from the curve fittings	Maximal inhibition of L-proline uptake (%) found for the given concentration applied (in brackets)	LDH release (% of initial cellular LDH activity)
L-alanine	0.57	111	90 ± 4 (5 mM)	1 ± 1
L-asparagine	0.065	64	64 ± 7 (5 mM)	0 ± 0
L-methionine	0.31	87	86 ± 9 (5 mM)	1 ± 1
L-serine	0.16	77	76 ± 9 (5 mM)	1 ± 2
L-threonine	1.22	87	58 ± 10 (5 mM)	1 ± 1
L-valine	0.17	–	50 ± 5 (5 mM)	4 ± 2
L-4-hydroxyproline	1.17	98	85 ± 13 (5 mM)	6 ± 4
L-homocysteine	0.17	86	101 ± 15 (5 mM)	3 ± 5
L-4-chlorophenylglycine	0.034	55	78 ± 13 (0.5 mM)	3 ± 2

Shown are data for the substances that were found to significantly inhibit L-proline uptake by astrocytes (Figs. 6 and 8). The data are derived from 3 independent experiments that had been performed on independently prepared cultures. The data for the concentration-dependencies of the inhibitors were fitted by a 4-parameter logistical model and analyzed for the concentrations allowing half-maximal inhibition of L-proline uptake (EC_50_) and for the maximally achieved inhibition. In addition, for the highest concentrations of the inhibitors applied, the corresponding inhibition and the extracellular LDH activity found for this condition are given. The data obtained for L-valine did not allow a reliable calculation of the maximal inhibition using the logistical model

**Fig. 7:**
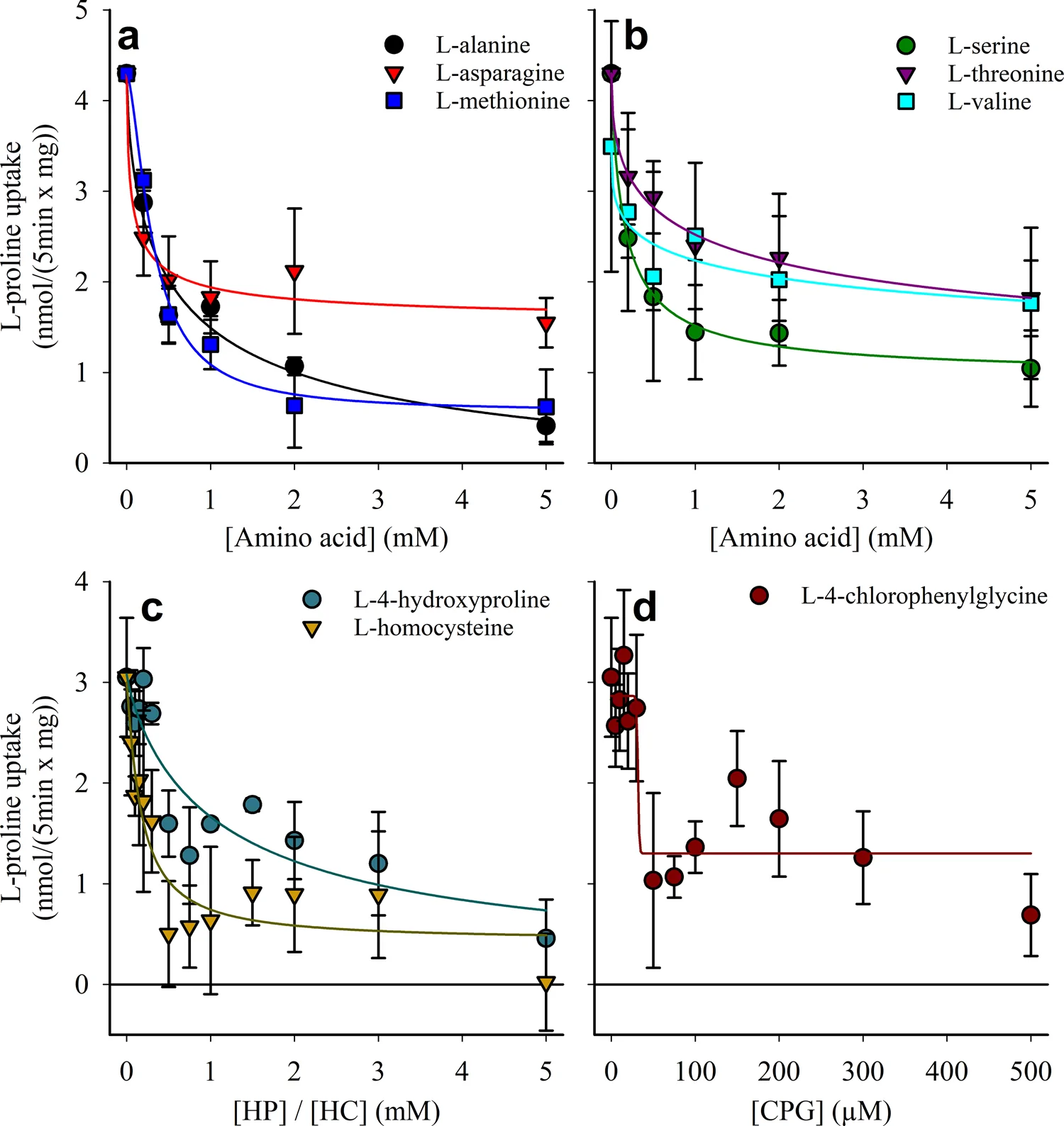
Concentration-dependent effects of amino acids and inhibitors of amino acid transporters on the astrocytic uptake of L-proline. Primary astrocytes were pre-incubated in PB with N-PPG for 1 h, followed by a 5 min main incubation with 0.2 mM L-proline and the indicated concentrations of L-alanine, L-asparagine, L-methionine, L-serine, L-threonine, L-valine, L-4-hydroxyproline (HP), L-homocysteine (HC) or L-4-chlorophenylglycine (CPG). After the incubation, the cellular proline levels were determined and used to calculate the specific proline uptake velocities. For experiments with L-alanine, L-asparagine, L-methionine, L-serine and L-threonine the initial cellular LDH activity was 137 ± 20 nmol/(min x well) and the initial cellular protein content was 128 ± 12 µg/well. For experiments with L-valine, HP, HC and CPG the initial cellular LDH activity was 189 ± 60 nmol/(min x well) and the initial protein content was 154 ± 34 µg/well. The data represent means ± SD of values obtained in three independent experiments that had been performed on independently prepared cultures. Dose-response curves were fitted using a 4-parameter logistical model and used to calculate EC_50_ values which are given in Table 3. Additionally, the extracellular LDH activities were measured and have been included in Table 3 for the given highest concentration of competitor or inhibitor applied

The inhibitory potential of the 6 amino acids that in excess had been found to lower astrocytic L-proline accumulation (Fig. 6a) was further investigated by determining the concentrations of the respective amino acids that lowered astrocytic L-proline (0.2 mM) accumulation by 50% (EC_50_) and the maximal inhibition found in the presence of 5 mM of the respective competing amino acid (Table 3). All six tested proteinogenic L-amino acids slowed down the astrocytic L-proline accumulation in a concentration-dependent manner (Fig. 7a, b). Strongest inhibition was found for concentrations of 5 mM (Fig. 7a, b; Table 3) with values that were quite similar to those found for concentrations of 2 mM (Figs. 6 and 7). The calculated EC_50_ concentrations for the half-maximal inhibition of L-proline accumulation revealed that L-asparagine had the lowest EC_50_ value (around 65 µM), followed by L-serine, L-valine, L-methionine, L-alanine and L-threonine (Table 3).

**Fig. 8:**
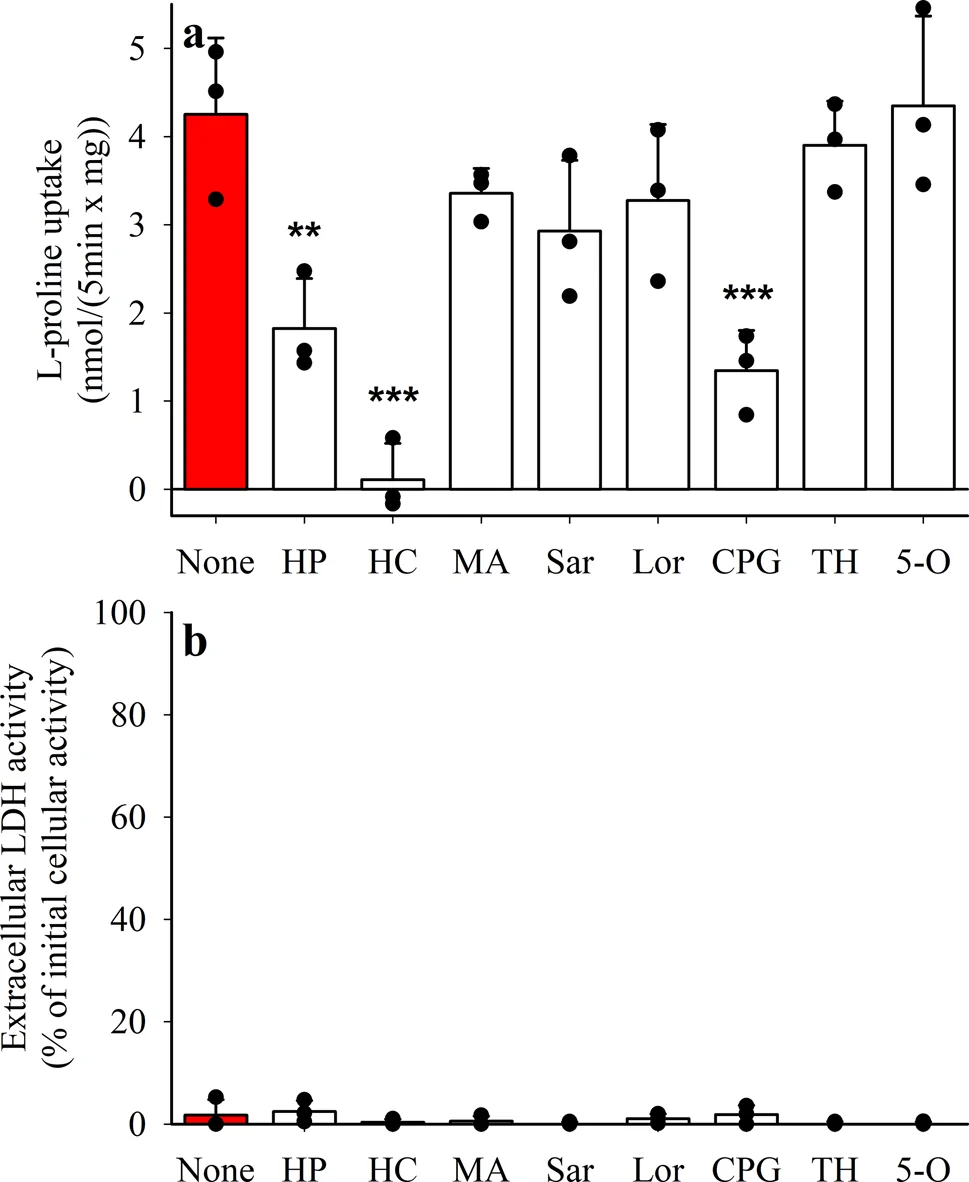
Test for the effects of non-proteinogenic amino acids and inhibitors of amino acid transporters on the uptake of L-proline by cultured astrocytes. The cells were pre-incubated in PB with N-PPG for 1 h, followed by a 5 min main incubation with 0.2 mM L-proline in the absence or the presence of 3 mM of either L-4-hydroxyproline (HP), L-homocysteine (HC), MeAIB (MA), sarcosine (Sar), THFA (TH) or 5-Oxo (5-O) or of 0.3 mM of loratadine (Lor) or L-4-chlorophenylglycine (CPG). After the incubation, the cellular proline levels were determined and used to calculate the specific proline uptake velocities (**a**). Additionally, the extracellular LDH activities were measured (**b**). The initial cellular LDH activity was 138 ± 16 nmol/(min x well) and the initial protein content was 119 ± 13 µg/well. The data represent means ± SD of values obtained in three independent experiments that had been performed on independently prepared cultures. Significance of differences of data (ANOVA) compared to data obtained for incubations without competitors or inhibitors (None) is given as **p < 0.01 and ***p < 0.001

In addition to the proteinogenic amino acids, the non-proteinogenic amino acids L-4-hydroxyproline (HP) and L-homocysteine (HC) as well as sarcosine (methylglycine) were investigated for their potential to interfere with L-proline uptake in astrocytes. In 3 mM concentrations, astrocytic accumulation of 0.2 mM L-proline was significantly lowered by HP and HC, but not by sarcosine (Fig. 8a). For both HP and HC their inhibitory potential depended on the concentrations applied and was saturable (Fig. 7c). Fitting the dose-response curves revealed that HC (EC_50_ = 0.17 mM) had a stronger potential to lower astrocytic L-proline accumulation than HP (EC_50_ = 1.17 mM) (Table 3).

### Inhibition of L-Proline Uptake by Known Inhibitors of Amino Acid Transporters

Several transporters are expressed in astrocytes that have been reported to accept L-proline as substrate (Table 1). In order to test for a potential contribution of such transporters, the uptake of L-proline (0.2 mM) into primary astrocytes was investigated in the absence or the presence of known inhibitors of such L-proline transporters. Among the tested inhibitors, only L-4-cholorophenylglycine (CPG) lowered efficiently the astrocytic L-proline accumulation by around 70–80% (Fig. 8) with a rather low half-maximal inhibitory concentration (EC_50_ = 34 µM) (Fig. 7d; Table 3). In contrast, the presence of the transporter inhibitors loratadine and MeAIB did not significantly lower astrocytic L-proline accumulation (Fig. 8a). Also the proline analogues THFA and 5-Oxo, which are inhibitors of proline dehydrogenase [51–53], had no effect on the L-proline accumulation, suggesting that their inhibitory effect on L-proline consumption [22] is mediated by inhibition of proline dehydrogenase and not by inhibition of L-proline uptake.

## Discussion

L-Proline is efficiently consumed by starved primary astrocytes to maintain high cellular ATP levels [22, 23]. As this process requires uptake of L-proline, we have investigated the mechanisms involved in the uptake of L-proline for cultured primary astrocytes by determining the cellular L-proline accumulation in the presence of the ProDH inhibitor N-PPG which prevents cellular metabolism of the internalized L-proline [22]. L-proline was efficiently accumulated by cultured astrocytes in a temperature-, time- and concentration-dependent manner which was characterized by a rapid initial accumulation during the first minutes that slowed during longer incubations and reached for all concentrations applied maximal values after around 60 min. Analysis of the initial rapid L-proline accumulation revealed a concentration-dependency that followed apparent Michaelis-Menten type kinetics, with a K_M_ value of 2 mM and a V_max_ value of 13 nmol/(min x mg) for 5 min incubations. This V_max_ value for L-proline accumulation was much higher than the reported maximal velocity for L-proline consumption by astrocytes (1.7 nmol/(min x mg)) [22], suggesting that the cellular metabolism and not the uptake limits L-proline consumption by astrocytes.

The cytosolic concentrations calculated from the specific cellular L-proline contents determined after 60 min incubations strongly exceeded the initially applied extracellular concentrations, clearly demonstrating that L-proline is accumulated by astrocytes against a concentration gradient by an active transport process. The transporters expressed in astrocytes that have been connected with L-proline transport depend all on the presence of sodium ions (Table 1). Omission of Na^+^-ions from the incubation medium revealed that a saturable Na^+^-dependent and a non-saturable Na^+^-independent process contribute to L-proline accumulation by cultured astrocytes. The Na^+^-dependent component of the cellular L-proline accumulation was found to have a K_M_ value of 1 mM. This K_M_ value is substantially higher than the reported physiological serum concentration of L-proline that range between 50 µM and 450 µM [8, 54–57], but will enable the astrocytes to take up L-proline with a velocity that is proportional to the extracellular L-proline concentration. In addition, a high K_M_ value will provide sufficient capacity to increase the transport rate under conditions of elevated L-proline concentrations that have been reported to reach the low millimolar range in pathological conditions [8, 58].

For extracellular L-proline concentrations of up to 1 mM, more than 90% of the detected L-proline accumulation was Na^+^-dependent, while hardly any D-proline accumulation was found. This suggests that a Na^+^-dependent transporter that is specific for the L-isomer of proline mediates cellular accumulation in the low concentration range. The linear concentration-dependent L-proline accumulation found for Na^+^-free incubations came only into play for unphysiologically high concentrations of L-proline. A similar contribution of a saturable Na^+^-dependent and a linear Na^+^-independent amino acid transport has been reported for the uptake of L-serine in rat astrocytes [59]. The linear concentration-dependency of the Na^+^-independent accumulation of L-proline and D-proline (data not shown) and the lack of stereospecificity suggest that this process is mediated by some type of diffusion. This view is supported by similar specific cellular proline contents that were determined after 60 min incubations with either 10 mM L-proline or D-proline in the absence of Na^+^ (data not shown). A diffusion-mediated transport of L-proline has previously also been reported to contribute to the transport of L-proline over the blood-brain barrier [60] and to the L-proline uptake into isolated rat brain capillaries [61]. Permeation of a L-proline molecule which exists as dipolar ion at physiological pH through the lipid bilayer appears to be rather unlikely, suggesting that a protein channel may contribute to the uptake of L-proline at high concentrations. However, proline in higher concentrations has also been shown to form dimers [62]. It remains to be elucidated whether the electrostatic interaction between the carboxylate and ammonium groups of two proline molecules that form a proline dimer may lower the polar properties of the dissolved amino acid, thereby increasing the potential to permeate the lipid phase of a plasma membrane.

A total of five different Na^+^-dependent transporters that have been connected with the transport of L-proline were shown to be expressed in astrocytes (Table 1). Substrate competition and inhibitor studies were performed to narrow down which of the potential L-proline transporters may contribute to the observed L-proline uptake in astrocytes. The determined K_M_ value for astrocytic L-proline uptake of 1 mM is quite similar to values previously reported for L-proline uptake by ASCT1 (0.7 mM; [28]) or ATA2 (around 0.8 mM; [25, 33]), but substantially higher than reported K_M_ values of L-proline uptake by other transporters (Table 1). In addition, results from substrate competition experiments demonstrated that only excess of the six proteinogenic amino acids L-serine, L-alanine, L-threonine, L-asparagine, L-valine or L-methionine lowered L-proline uptake, but not additional amino acids that are known to be substrates of some of the potential astrocytic L-proline transporters (Table 1). This spectrum of competing amino acids for L-proline uptake matches to the reported potential of L-proline, L-alanine, L-threonine, L-asparagine or L-valine to inhibit ASCT1-mediated L-serine uptake in primary rat astrocytes [63], and argues against a strong contribution of the rather substrate specific Imino transporter in astrocytic L-proline uptake (Table 1). While the inhibition of astrocytic L-proline uptake by L-hydroxyproline (HP) would be consistent with the involvement of the transporters ASCT1, B^0^AT2 and Imino (Table 1), the strong inhibition by L-homocysteine (HC) would be rather specific for ASCT1 as this transporter is (among proline-accepting transporters) to our knowledge the only one known to transport HC [64, 65]. Finally, the strong contribution of ASCT1 in astrocytic L-proline uptake is confirmed by the concentration-dependent inhibition of L-proline accumulation by the ASCT1 inhibitor CPG [18], while inhibitors of other potential L-proline transporters such as loratadine for B^0^AT2 and MeAIB for Imino and the system A member ATA2 (Table 1) did not significantly lower L-proline accumulation. In addition, the low concentration of CPG that half-maximally inhibited L-proline uptake (34 µM) was rather similar to the half-maximal inhibition (around 25 µM) found for ASCT1-mediated L-serine uptake into cultured astrocytes [66]. Together, these data suggest that predominately ASCT1 contributes to the L-proline uptake by cultured astrocytes. Ample evidence has been presented in the literature showing that ASCT1 is expressed in astrocytes in vitro and in vivo. Several groups have reported the expression of ASCT1 for cultured astrocytes from rat brain as well as the involvement of this transporter in astrocytic amino acid uptake [30, 59, 63]. In addition, ASCT1 has frequently been shown to be expressed in astrocytes in brain by RNA expression analysis and by immunocytochemical staining of brain sections derived from mouse [67, 68], rat [69] and human [70] brain, demonstrating that this transporter is expressed in astrocytes in brain and could contribute to astrocytic L-proline uptake in vivo.

As most of the amino acid substrates of ASCT1 as well as the ASCT1 inhibitor CPG were even in high concentrations unable to completely inhibit astrocytic L-proline uptake, it has to be concluded that in addition to ASCT1 one or more additional transport processes contribute to some low extend to the L-proline uptake under the conditions studied. The characteristics of the ASCT1-independent L-proline transport and the identity of the transporters involved remain to be elucidated. Our inhibitor studies did so far not hint towards a transporter among those reported to be expressed in astrocytes (Table 1) that is likely to contribute to the ASCT1-independent astrocytic L-proline uptake. Further studies could be performed to explore whether the four additional transporters that have been connected with L-proline transport and reported to be expressed in astrocytes (B^0^AT2, Imino, ATA2, SNAT5) are expressed in the rat astrocyte cultures used for our study, for example by RT-PCR analysis, immunocytochemical staining and/or Western blotting. However, it should be considered that also other L-proline transporters that have so far not been investigated in detail concerning an expression in rat astrocytes may be expressed in the cultures used and could participate in the ASCT1-independent L-proline uptake. An ASCT1-knockdown approach could be a useful strategy to investigate the contribution of other transporters as well as the characteristics of the residual ASCT1-independent L-proline uptake.

In summary, primary rat astrocytes actively take up and accumulate extracellular L-proline in a concentration-dependent, saturable and Na^+^-dependent manner. This uptake is stereospecific and temperature-dependent. Results from substrate competition and inhibitor experiments demonstrate that predominately the neutral amino acid transporter ASCT1 is responsible for the observed rapid accumulation of L-proline in astrocytes and that other transport processes contribute to a minor extend to the L-proline uptake under the conditions studied.

## Supplementary Information

Below is the link to the electronic supplementary material.

Supplementary Material 1

## Data Availability

Enquiries on original data should be directed to the corresponding author.
