# Supplementary material for: Uptake of L-proline by Cultured Primary Rat Astrocytes
Source: Neurochem Res. 2026 Sep 30;51(5):279. doi: 10.1007/s11064-026-04886-5 (PMC13627194; doi:10.1007/s11064-026-04886-5)
Supplement: Supplementary file 1 [file 11064_2026_4886_MOESM1_ESM.pdf]

Uptake of L-proline by cultured primary rat astrocytes

Paul Spellerberg and Ralf Dringen

Supplementary Information

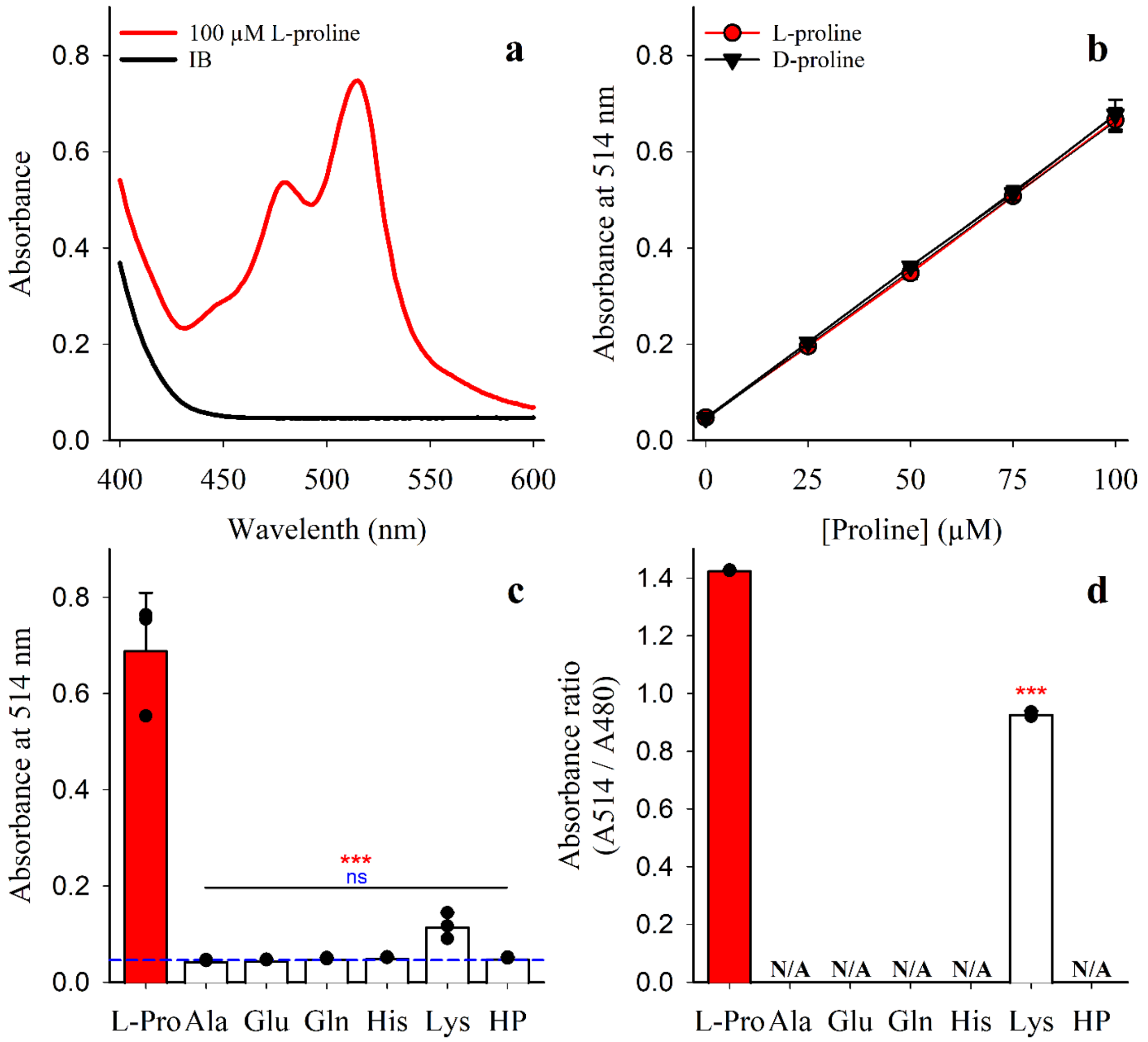

Figure S1: Quantification of L- and D-proline by a ninhydrin-based colorimetric assay according to Harders et al (2024). 100 µL of an amino acid-containing sample in incubation buffer were mixed with 200 µL 1.25 % (w/v) ninhydrin in glacial acetic acid and incubated at 100°C for 30 min before the absorbance spectra or the absorbance at the given wavelengths were measured. Panel a shows representative spectra for the amino acid-free incubation buffer and for 100 µM L-proline that had been dissolved in IB. Panel b shows the concentration-dependent linear increase in absorbance at 514 nm for the ninhydrin complexes formed in the presence of L- and D-proline. Panel c shows the absorbance obtained for reactions of ninhydrin with 100 µM of L-proline compared to the absorbance at 514 nm determined for 100 µM of L-proline, L-alanine (Ala), L-glutamate (Glu), L-glutamine (Gln), L-histidine (His), L-lysine (Lys) or L-4-hydroxyproline (HP). Panel d shows the proline-specific characteristic ratio of the absorbances of the ninhydrin complex of 514 nm and 480 nm after subtraction of the baseline of the respective absorbance for the given substrates. The data in panels b, c and d represent means ± SD that had been obtained in three independent experiments. Significance of differences (ANOVA with Bonferroni post-hoc test) of the values obtained compared to the values determined for L-proline (red symbols) or compared to the values for the amino acid-free controls (blue line in panel c) are indicated as ***$p<0.001$ or as not significant (ns). N/A = not applicable due to an absorbance of 0 at 480 nm.
